# The Diet in Typhoid Fever1Read at the forty-first annual meeting of the Medical Association of Central New York, held at Buffalo, October 27, 1908. — Edited by William C. Krauss, M. D.,

**Published:** 1909-02

**Authors:** I. Harris Levy

**Affiliations:** Syracuse, N. Y.; Associate Professor of Clinical Medicine, College of Medicine, Syracuse University; Visiting Physician to The Hospital of the Good Shepherd


### The Diet in Typhoid Fever.[1]

By I. HARRIS LEVY, Ph.B., M.D., Syracuse, N. Y.
Associate Professor of Clinical Medicine, College of Medicine, Syracuse University;
Visiting Physician to The Hospital of the Good Shepherd.

THE diet in typhoid fever, we are told by all the textbooks should be liquid—mainly milk. It is fair to raise the question, why was this diet so generally adopted? Were typhoid patients formerly fed solid food, and was it found to lead to a high death rate or to serious complications, and did a starvation or fluid diet reduce them? No, such is not the history of the liquid diet. On the contrary, it is rather a survival. It dates from the antiphlogistic days when fevers were believed to be due to increased action of the heart and arterial system. To counteract this asthenic condition, it was considered necessary to puke, purge, bleed, and starve. Such was the treatment of all fevers.

With the dawn of the modern era, and the decline of the antiphlogistic pathology, our treatment of fevers changed. We no longer bled and purged, but continued the starvation diet.

---

1. Read at the forty-first annual meeting of the Medical Association of Central New York, held at Buffalo, October 27, 1908.—Edited by William C. Krauss, M. D.,

While the milk diet is strictly speaking not a starvation diet, yet, since it does not supply more than half the necessary calories of energy needed by a typhoid patient, it leads to considerable loss of strength and weight.

The advocates of the fluid diet claim that during typhoid, the digestive organs are impaired, and consequently solid food is badly borne: that milk is the most easily digested food—being a fluid it does not irritate the ulcers in the bowels; that solid food does irritate them, exposing the patient to the dangers of hemorrhage and perforation: and that relapses are common when solid food is allowed too early.

But despite these arguments, during the past ten years, a number of articles have been written by eminent clinicians both in America and abroad protesting against a liquid, and advocating a much more liberal diet in typhoid fever. They show that in actual practice solid food does not increase the death rate and complications, but, on the contrary, it seems to reduce them. Besides, the patients are much more comfortable, there is less loss of weight and strength, and the convalescence is shortened. Also, when the patient is fed solid food throughout the disease, there is practically no danger of over-eating during early convalescence, nor is there any need of obtaining food surreptitiously.

Let us examine the claims of the opposing advocates. Is digestion greatly impaired during typhoid fever? The experiments of von Hoesslin, Leyden and Klemperer, Puritz, Folin, and others show conclusively that the average typhoid patient absorbs from his intestines almost as completely as does the healthy individual. Only in the severest cases is the absorption materially decreased. In the average case, without profuse diarrhea, the digestion of carbohydrates, proteids, and fats is within 10 or 15 per cent. of the normal. Folin found the absorption of carbohydrates practically normal.

Is milk a perfect food for typhoid patients? It might be supposed that milk being a liquid would readily pass into the intestines. As a matter of fact, it is only a liquid outside of the body. In the stomach it soon becomes coagulated, and forms a tough leathery mass, which offers great resistance to the digestive juices. It has been experimentally demonstrated in the healthy individual, that a glass of milk does not entirely leave the stomach until two hours have elapsed from the time of swallowing it. A pint of milk requires no less than three and one-half hours for its complete disposal. After milk leaves the stomach, its digestion is completed in the intestines. When it is not well digested, as frequently happens, curds of varying size appear in the stools. Physiologists also tell us, that when milk is given by itself as the exclusive diet to adults in a state of health, it is not very well absorbed—worse indeed than any other animal food. As Hutchinson says in his Manual on Dietetics: "Even under the most favorable conditions, only about 90 per cent. of the available potential energy contained in the milk ever reaches the blood. The rest escapes from the body as unabsorbed waste." Milk is much better absorbed when it forms part of a mixed diet. Wait found that when milk alone was taken that 92.1 per cent. of the proteid and 86.3 per cent of the carbohydrate were digested. On the other hand, when the diet consisted of bread and milk 97.1 per cent. of the proteid and 98.7 per cent. of the carbohydrate were utilised. There are other objections to the milk diet. Some people dislike it, others cannot take it without upsetting the stomach. When badly borne, it is the worst of foods. Milk also may produce flatulence and tympanites. The latter frequently complicates typhoid fever, and when it does, it demands a change in the diet. Likewise the presence of curds and coagula in the stools demands a decrease in the quantity, or a complete withdrawal of the milk. Milk is a good culture medium for germs, and in children we know that it may cause the worst kind of intestinal indigestion. When the milk diet is employed in typhoid, it is customary to allow the patient about two quarts in the 24 hours—a glass every two hours. Two quarts of milk furnish about 1,300 calories of energy. A typhoid patient requires about 2,800 calories, more than double the amount furnished by the milk. Consequently, an adult loses from ten to sixty pounds during the course of the fever.

The amount of inorganic salts in the milk may also be detrimental during the later stages of the disease. Wright and Knapp have suggested that the calcium salts by increasing the coagulability of the blood favor thrombosis.

Can the body loss be prevented during fever by a more liberal diet? A number of experiments have been carried out to settle this point. Some loss is likely to occur even when a full diet is allowed. Shaffer and Coleman of Bellevue Hospital undertook to determine the extent to which it was possible to limit the loss of body protein. They fed their patients large amounts of carbohydrate in the form of milk sugar. They say:

Our results show that the febrile loss of body protein may be retarded and even wholly prevented or compensated for. But the results show likewise how difficult this is to accomplish. It was only when we gave 60 or 70 or even 80 calories per kilogram—between 3,000 and 4,000 calories in the twenty-four hours—that the greatest sparing was observed. The important point is however, that it is possible to give typhoid patients such liberal diets without, so far as our experience shows, producing any harmful results, but, we believe with decided benefit.

Does milk minimise the dangers of hemorrhage and perforation, and does solid food increase them? It is argued, milk being a fluid causes less irritation, while solid food coming in contact with the ulcers tends to irritate them. Here there is a double fallacy. It has already been pointed out that milk in reality is not a liquid food, that it forms leathery curds, and unless well digested passes through the bowels in hard cheesy masses. On the other hand, solid food, with the exception of cellulose, seeds and the like, is in a liquid state by the time it reaches the small intestine. It is not the condition of the food as it enters the mouth, but its condition when it reaches the bowels that is important.

Too much stress has been laid on the intestinal manifestations of typhoid. In our treatment we have centered our attention on the ulcers and the bacteria. Hence the use of the various intestinal antiseptics for which such marvelous results have been claimed. The newer investigations, however, have shown that typhoid in its early stages at least, is a bacteremia. Blood cultures show the presence of the Eberth bacillus at some stage of the disease in practically all of the cases. A certain number of cases of typhoid run their course without any intestinal lesions. There is a typhoid with local manifestations limited entirely to the gallbladder. The severity of the diarrhea is likewise no indication of the number or the depth of the ulcers. Severe diarrhea may be present with superficial ulcers or even with intact bowel, as in a case reported by Dr. Bryant. On the other hand, diarrhea may be absent with deep ulceration. It has by no means been demonstrated that food is the cause of an ulcer perforating. It is evident that large irritating masses might cause the thin bowel to give way. But no one would advocate such a diet for a typhoid patient. Perforation occurs in typhoid irrespective of the diet—in the starved case as well as in the fed. As Bushuyev says: "Perforation depends more on the nature of the ulcer than upon the diet."

The same fear of perforation led us to withhold food by mouth for some days following hemorrhage in gastric ulcer. This was a universal rule. But since Lenhartz has shown that our fears were groundless, we must modify our ideas. He feeds his gastric ulcer patients by mouth from the start, and his results are better than under the old treatment. Lambert, Minkowski, and Wirsing have followed his dietary and report favorable results. Wirsing states that his results were best in the severe cases. Again, recent autopsy investigations in pulmonary tuberculosis show that tubercular ulcers are very common in the ileum. Yet forced feeding is the rule in tuberculosis and no one fears a perforation. Barrs of Leeds, who is an advocate of liberal feeding in typhoid fever says:

> It is difficult for me to conceive any method of treatment more calculated to delay healing and so favor perforation than the prolonged period of starvation which the plan of fluid feeding, still advocated as essential for 10 or 14 days after the febrile state has come to an end, adds to the devastating effect of pyrexia, and limited feeding of the disease itself. The conditions favorable to healing are the same for the small intestine as for any of the external parts of the body, and no surgeon, I imagine, would expect to see wounds undergoing repair in the shortest possible time in a patient living on fluids alone.

Does a liberal diet in typhoid fever relapse? A relapse sometimes does occur when a sudden change is made in the diet. But it is a question if it is more than a coincidence. Relapses occur on a fluid diet when no change has been made. On the other hand, they occur only in a small percentage of the cases when solid food has been given throughout the disease. Relapse as we now understand it, means re-infection. It is doubtful if a true relapse ever is due to diet alone. Food should no more cause a true re-infection than the original attack.

It is argued that solid food liberating a large amount of energy increases the fever. During typhoid an elevation of temperature sometimes occurs when a sudden change is made either in the quantity or quality of the food—the so-called *febris carnis* or food fever. Its explanation is not clear. Some consider it akin to the fever caused by excitement. Others regard it as due to the liberation of a large amount of energy. The heat regulating center accustomed to the low diet cannot easily accommodate itself to the changed conditions, and as a result is thrown out of equilibrium. When it does occur, it is of brief duration and of minor importance—very different from the true relapse.

Pawlow and his followers have shown the effect of appetite on the digestive secretions. When food is forced or not relished a poor gastric juice is secreted. The diet should therefore not be monotonous but varied. The diet also has a marked influence on the secretion of bile. On a starvation diet very little, if any, is secreted. The bile stagnates in the gallbladder and is then likely to be infected by the intestinal flora. Flexner found the Eberth bacillus in the gallbladder in 50 per cent. of his typhoid cases coming to autopsy. The germs may produce a complicating cholecystitis or may remain quiescent for years. The typhoid bacillus has even been found in gallstones. Pawlow has shown that some foods produce a greater flow of bile than others. Meat is the most powerful excitent of this secretion. The more proteid the food, the more fluid the bile and the better the drainage of the gallbladder, and, consequently, the less likelihood of infection.

Theoretical arguments, however, cannot settle a question of this nature. "The proof of the pudding is in the eating," and so here, the practical results must decide. It must be demonstrated that a liberal diet not only does not increase the dangers of typhoid, but that it has positive advantages. A number of careful clinicians in various parts of the world have urged a more liberal feeding—basing their claims on their results.

Among the first to try the liberal diet was Bushuyev of the Military Hospital of Kiev. He allows his patients a very liberal diet. During 1895-96, he treated 80 patients on this diet, while a colleague treated 74 on a fluid diet, chiefly milk. His mortality was 10 per cent., while that of his colleague was 12.1 per cent. During 1897, he treated 318 cases on a solid diet with a mortality of 8.2 per cent. The average hospital mortality for typhoid for the ten previous years was 12.4 per cent. Of 1,100 cases treated in Russian hospitals on this diet there were but 69 deaths, a percentage mortality of 6.3 per cent. Bushuyev says that:

Under this regime the general condition of the patient is incomparably better than when kept upon an exclusively milk diet. The common complaints are scarcely ever heard. At breakfast, dinner and supper the patients are uncommonly wide awake; even those who are very ill; sit up in bed, beg for food, and eat with much satisfaction; only a few have to be fed by nurses. If one observe the patients at meal times he wholly forgets that these individuals are seriously ill, with temperature above 39 C. The military authorities are amazed on visiting a typhoid ward at the time of breakfast or dinner, in as much as they see almost no typhoids.

The observation of the late Prof. M. T. Chudnovsky that the typhoid state disappears under a full diet, is confirmed. During the first hours in the ward the patient lies in a motionless condition, failing to answer questions and refusing food. But if one succeed in some way or other in persuading him to eat a bit of meat, or cutlet or an egg, he immediately begins to show some interest in the surroundings. In a few days, often within a day, no trace of the typhoid condition remains. Unfortunately, it is impossible to persuade all typhoid patients to eat. Attempts at forced feeding cause vomiting.

Thayer who abstracted the article for Progressive Medicine (1899), comments as follows:

Is it not more than likely that many cases of typhoid fever suffer from too restricted a diet? Bearing in mind the long course of fever through which a patient must pass, the dangers to which he is exposed, not only from exhaustion and the accidents peculiar to typhoid fever, but especially from the various secondary infections to which he is so easy a prey at the end of his long period of fever and fasting, our main object should be to keep up his general nourishment by every means in our power. Obviously, if a more liberal diet than that afforded by the purely liquid regimen could be assimilated, the patient's strength would hold out materially better. In diphtheria or pneumonia or febrile tuberculosis, do we not make every effort to induce the invalid, to take as much as he can bear of a simple, easily absorbable, and nourishing diet? And yet in typhoid fever we are restrained by a vague fear that any departure from the customary regimen is for some reason or other, dangerous. What ground have we for the fear? The ordinary answer is the quotation of a case where, after a long course of fever with a much restricted diet some indiscretion has produced a sudden rise of temperature with alarming symptoms. An indiscretion in diet may produce symptoms in any condition of severe physical exhaustion, but the reviewer has never seen anything to suggest that it is more common in typhoid fever than in any other similar condition.

Barrs in England also early advocated a solid diet in typhoid. In 1897 he reported 31 cases with 3 deaths. In these 3 fatal cases, he was never able to give solid food. There were no deaths in the cases receiving solid food early. He concluded his paper by saying:

I have not hitherto urged any reason for the plan which I advocate beyond the fact that it involves the least departure from the normal line of health. But I am prepared to urge that it is a method which is likely to modify favorably the death rate of typhoid fever, to shorten convalescence, to diminish the risks of complications and sequelæ and generally to make typhoid fever, a less formidable and more manageable disease than it is under our present standard methods of treatment.

Marsden, in 1900, reported 200 cases fed on bread and milk, custard, fish with mashed potato, chicken, bread and butter, and minced meat. He says:

I think from a review of these 200 cases it must be concluded that a careful system of dieting, such as I have mentioned, has no injurious consequences, and when one considers the benefits obtained—viz., more rapid recovery, diminished risk of surreptitious feeding with possible harmful substances, lessened tendency to bolt food and finally lessened tendency to asthenic complications, as post-typhoid anemia, gangrene, and the like, one must admit that there is no justification for resisting a craving appetite in the manner at present in vogue.

In America the earliest and strongest advocate of a liberal diet in typhoid has been Shattuck of Boston. During 1886,-1893, he treated 233 patients on an exclusively milk diet with a mortality of 10 per cent. From 1893 to 1902, he treated 246 patients on a liberal diet and the mortality was but 8.45 per cent. While treating his patients in the orthodox manner, he says:

I closed my ears to the clamors of adults, and my eyes and heart to the tears of children, as I now believe, unnecessarily. He further says, "One of the things which set me thinking on this question of the diet in typhoid was the favorable course run by several acute febrile cases for whom I ordered a full diet because they were weak: believing at the time of so doing that typhoid could be excluded, but being forced to the conclusion later that only typhoid fever could explain the whole course of the disease. These patients did perfectly well, were happier and convalesced more rapidly than my recognised typhoid cases fed exclusively on milk.

Shattuck's diet consists:

1. Milk, hot or cold, with or without salt, diluted with lime-water, soda water, apollinaris, vichy; peptogenic and peptonised milk; cream and water, milk with white of egg, slip, buttermilk, koumyss, matzoon, milk whey, milk with tea, coffee and cocoa.

2. Soups: Beef, veal, chicken, tomato, potato, oyster, mutton, pea, bean, squash, carefully strained and thickened with rice (powdered), arrow root, flour, milk or cream, egg, barley.

3. Horlick's food, Mellin's food, malted milk, carni-peptone, bovinine, somatose.

4. Beef juice.

5. Gruels. Strained oatmeal, crackers, flour, barley water, toast water, albumin, water with lemon juice.

6. Ice cream.

7. Eggs, soft boiled or raw, egg nog.

8. Finely minced lean meat, scraped beef, the soft part of raw oysters, soft crackers with milk or broth, soft puddings, without raisins, soft toast without crust, blanc mange, wine jelly, apple sauce and macaroni.

Fitz in a study of typhoid at the Massachusetts General Hospital for the 78 years ending with 1898, tabulates the mortality and complications in the various years, and under the changing treatment. Especially interesting and bearing on this subject of diet are the statistics for the years 1893 to 1898. He compares Shattuck's patients treated with a liberal diet and those of his colleagues restricted to milk and other fluids.

| | Mixed Diet Per cent. | Milk Diet Per cent. | Strained, Starchy and Proteid Fluids Per cent. |
|---|---|---|---|
| Mortality | 11.3 | 15.1 | 16.6 |
| Hemorrhage | 9. | 10.6 | 16. |
| Perforation | 3.4 | 2.8 | 1.8 |
| Relapses | 10.2 | 13.1 | 11.1 |

Fitz says in closing: "A considerable variety in diet may be permitted not only without detriment but also with possible benefit to the patient."

Kinnicutt follows Shattuck's dietary at the Presbyterian Hospital in New York. From 1900 to 1906, he treated 74 cases on this diet with a mortality of 12.16 per cent. He says: "By such a management I am convinced that much suffering may be avoided, prolonged, dissability materially modified, the dangers of secondary infections more efficiently met, and a more rapid convalescence effected."

Nichols, of Washington, who has written a splendid monograph on this subject, reports 40 cases of his own treated with a liberal diet with most gratifying results. He tabulates 1,518 cases treated in the various hospitals of the world on the mixed diet with 104 deaths, a mortality of 6.9 per cent.

Manges, LeFevre, Claytor, Morehouse, Hare, Smith and many others in America and abroad have also strongly urged an enlarged dietary in typhoid.

My experience with a mixed diet in typhoid dates from the fall of 1906. Since then, I have treated 24 patients sick with typhoid in the wards of the Hospital of the Good Shepherd and in private practice after the manner of Shattuck.

My first patient was a nurse. For the first two days she was on a fluid diet. She was exceedingly nervous and irritable. She absolutely refused to take milk. She was kept in bed with difficulty and did not sleep well. We anticipated a great deal of trouble, as in every way she promised to be a difficult patient to handle. I then allowed her bread and butter, custard, boiled rice and soft boiled egg, and later chicken and broiled steak. From the moment she was allowed solid food, there was a complete change in her behavior. The nervousness and irritability disappeared. She looked forward to her meals with a great deal of interest, and the subsequent course of the disease was uneventful. She left the hospital at the end of six weeks, and in two more she resumed her work as nurse, apparently none the worse for the illness. I must confess that for the first few days I watched the experiment with trepidation, and at all times it seemed intensely strange to see a typhoid patient with a temperature of 104 eating bread and butter, chicken and beefsteak with impunity.

All of my 24 patients so treated recovered. There was not a single case of perforation or severe hemorrhage. There were three relapses; otherwise, complications were conspicuous for their absence.

Not all of the patients are kept on the same diet, nor is there any attempt at forced feeding. The individual rather than the disease is treated. As soon as the patient admits that he is hungry and expresses a desire for food, it is allowed. The presence of fever in itself is never a contra-indication. The quantity of food is gradually increased and the effect watched, and if for any reason it is deemed advisable, the quantity as well as quality is changed.

Great care is necessary in making any material change in the regimen of a patient kept on a fluid diet for several weeks or more. The digestive organs, like the rest of the body, owing to the low diet is in a much reduced state, and cannot respond to any sudden demand made by an increase either in the quantity or the quality of the food. A rise in temperature may follow these indiscretions. Formerly such elevations of the temperature occurring during convalescence were supposed to mean that solid food was given too early, and that a relapse had occurred. In reality, it only meant that the unnecessarily weakened digestive organs could not resume their full functions without gradual training.

There is a marked difference in the appearance of the fed patient and those kept on a liquid diet. The former are more comfortable, the tongue is clean. There is an absence of the apathy and indifference so characteristic of the disease. The patients anxiously look forward to their meals, and do not exhibit the typhoid state. Physicians visiting the wards are surprised at the appearance of the patients, and admit that they never before had seen such comfortable looking typhoids.

Not only is the patient more comfortable during the course of the fever, but the period of convalescence is shortened. Having lost less, there is less to regain. The resistance of the patient being better, he is less likely to fall a prey to the secondary infections so common with typhoid. And lastly, the patient is able to resume his or her occupation much earlier—a consideration of no small importance especially to the working classes.

I am fully aware that in so variable a disease as typhoid it is dangerous to draw conclusions from a few cases. However, I am of the opinion that there is already sufficient evidence to warrant the belief that we have been responsible for considerable unnecessary suffering by adhering to too strict a diet in typhoid. A more liberal dietary apparently seems to diminish rather than increase the mortality and complications.

---

IN operating under local anesthesia in diabetic patients it is well to omit the use of suprarenal preparations, as in these cases it is liable to favor the occurrence of gangrene.—*International Journal Surgery.*

